# Total Synthesis of (–)-Anaferine: A Further Ramification in a Diversity-Oriented Approach

**DOI:** 10.3390/molecules25051057

**Published:** 2020-02-27

**Authors:** Elisa Bonandi, Giada Tedesco, Dario Perdicchia, Daniele Passarella

**Affiliations:** Università degli Studi di Milano, Via Golgi 19, 20133 Milano, Italy; giada.tedesco@oci.uni-hannover.de (G.T.); dario.perdicchia@unimi.it (D.P.)

**Keywords:** bis-piperidine alkaloids, anaferine, diversity-oriented synthesis, 2-piperidine ethanol

## Abstract

The piperidine ring is a widespread motif in several natural bioactive alkaloids of both vegetal and marine origin. In the last years, a diversity-oriented synthetic (DOS) approach, aimed at the generation of a library of piperidine-based derivatives, was developed in our research group, employing commercially available 2-piperidine ethanol as a versatile precursor. Here, we report the exploration of another ramification of our DOS approach, that led us to the stereoselective total synthesis of (–)-anaferine, a bis-piperidine alkaloid present in *Withania somnifera* extract. This natural product was obtained in 9% overall yield over 13 steps, starting from a key homoallylic alcohol previously synthesised in our laboratory. Therefore, the collection of piperidine-derivatives accessible from 2-piperidine ethanol was enriched with a new, diverse scaffold.

## 1. Introduction

**Figure 1:**
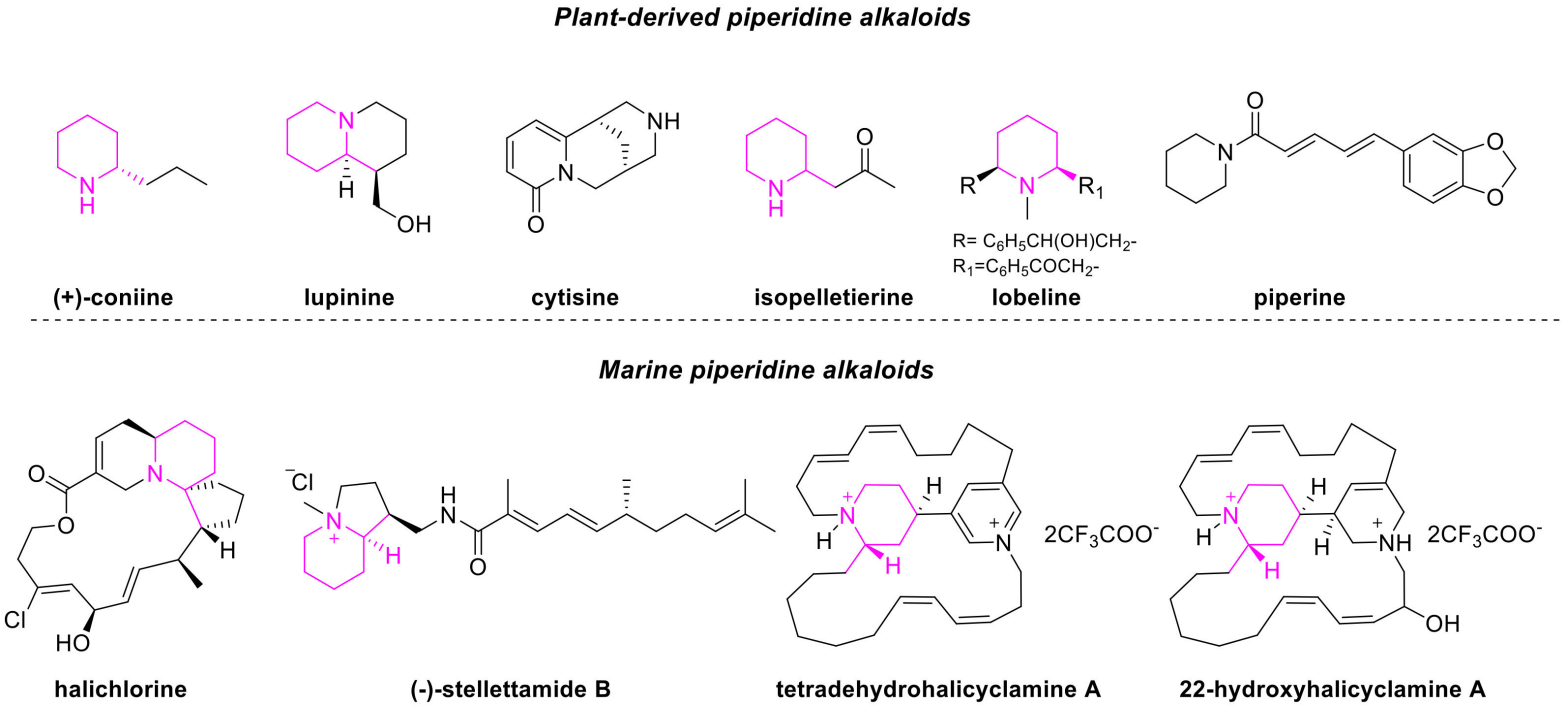
Structures of some representative natural piperidine alkaloids, from vegetal and marine origin. The fundamental α-substituted piperidine scaffold, shared by some of them, is highlighted in pink.

Piperidine alkaloids are widespread natural products in the plant kingdom, but they are also present in the animal one, in particular in marine microorganisms [1]. They are historically known as potent poisons and teratogens: some well-known examples include coniine (from hemlock), lupinine (from lupin flour) and cytisine, contained in *Laburnum* spp. pods [2]. These effects are mainly the result of the nicotinic acetylcholine receptors (nAChRs) desensitisation [3]. However, piperidine alkaloids are characterised also by a wide range of interesting biological properties, including anthelminthic (e.g., isopelletierine), antioxidant and neuroprotective activities (e.g., lobeline) and the abilities of increasing drugs bioavailability and of contrasting multidrug resistance (e.g., piperine, lobeline) [1]. The structures of the mentioned alkaloids are depicted in Figure 1.

In the marine kingdom, some representative piperidine-containing bioactive compounds, isolated mainly from sponges, are halichlorine (anti-inflammatory, cytotoxic) [4] and stellettamide B (antifungal and RNA-cleaving activities, from *Stelletta sp*. [5]) (Figure 1).

Also several bis-piperidine alkaloids, in which the two heterocycles are embedded in complex macrocyclic systems, are diffused in marine microorganisms. These compounds proved to possess antifungal, antimicrobial properties and cytotoxic activity against different cancer cell lines. Some examples belonging to this class are tetradehydrohalicyclamine A and B, 22-hydroxyhalicyclamine A, haliclonacyclamine A and F, arenosclerins D and E, madangamine F and neopetrosiamine A [6,7,8,9,10].

In this context, we realized that some of the cited natural products shared a common structural motif, constituted by a piperidine ring substituted in the 2-position, generating in this way a defined stereocenter (shown in pink in Figure 1).

**Figure 2:**
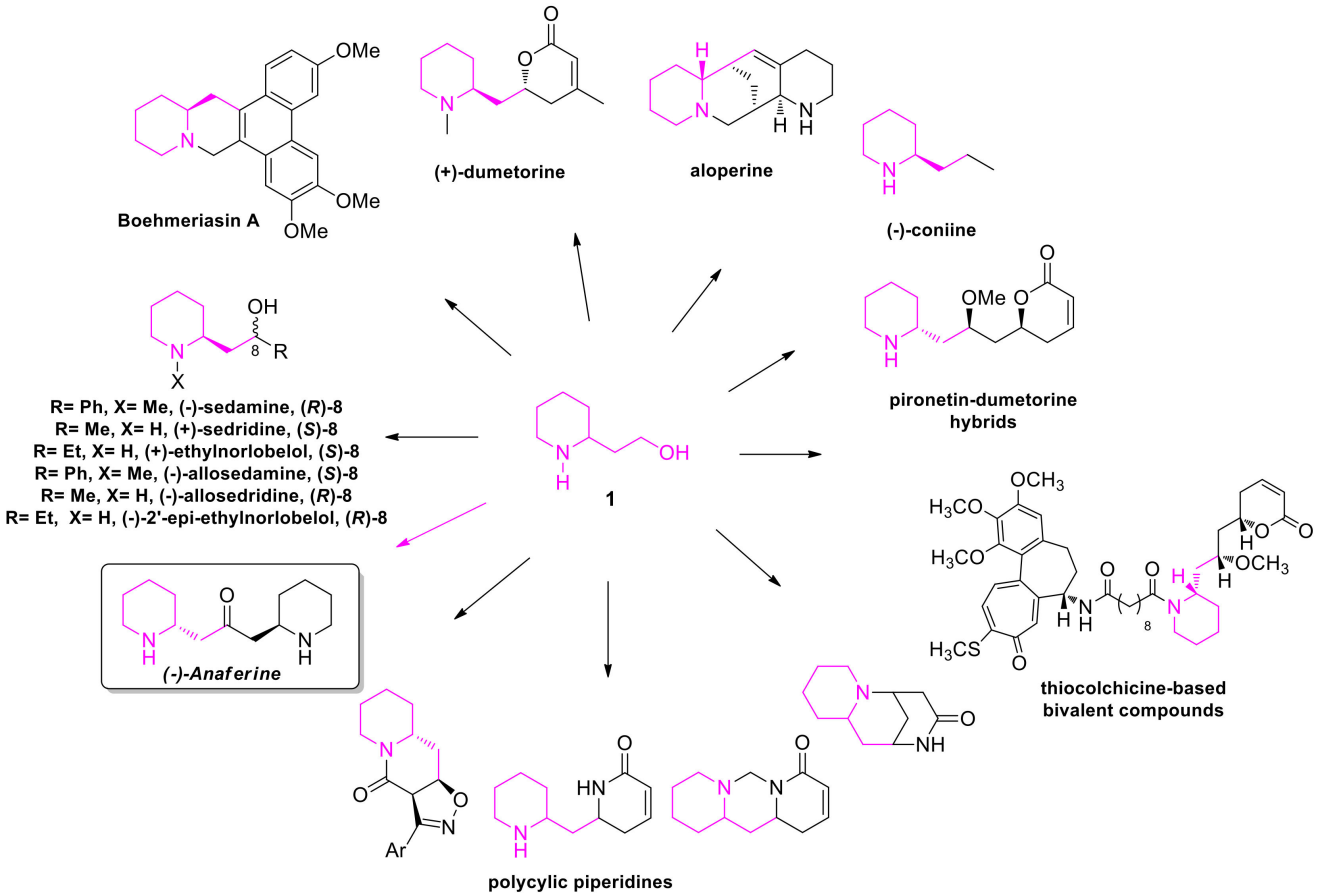
Structures of the representative members of our piperidine-based library, obtained through the DOS approach starting from 2-piperidine ethanol.

These features seemed quite familiar to us, because they characterised a library of compounds previously synthesised in our laboratory. In fact, the importance of the piperidine ring in bioactive natural products, prompted us to devise a diversity-oriented approach (DOS), aimed at the generation of a diversified library of derivatives containing this key heterocycle. In this project, the ideal starting point to achieve structural diversification proved to be the 2-piperidine ethanol (**1**), a cheap and commercially available racemic product that incorporates the fundamental highlighted 2-substituted-piperidine moiety. Our library is composed by natural products (such as aloperine, coniine, boehmeriasin A and sedum alkaloids [11,12,13,14]), and synthetic derivatives [15,16,17,18], as depicted in Figure 2.

We are currently trying to further enrich our library with new scaffolds. To this end, here we report the synthesis of (–)-anaferine starting from **1**, as a further ramification of our DOS approach. 

(–)-Anaferine is a bis-piperidine alkaloid, in which the two rings are connected through their C-2 atoms by a 2-propanone bridge. Being C2-symmetrical, this framework exists as two enantiomers and a meso-form. The diastereomeric mixture was isolated for the first time in 1962 [19] from the root extract of *Withania somnifera*, a plant widely exploited in the Ayurvedic medicine on the basis of several biological properties, including anti-inflammatory, anti-stress, immunomodulatory, adaptogenic, anticancer and neuroprotective activities [20]. ORD (optical rotary dispersion) analysis led to the determination of the absolute configuration of the two enantiomers: (–)-anaferine proved to be the (*R,R*)- enantiomer, and (+)-anaferine the (*S,S*)-one [21].

Most of the studies and the synthetic efforts on this topic, date back to the 70s, but in the last years this alkaloid has attracted once again the interest of the scientific community, thanks to *in silico* studies that suggested possible applications in the treatment of neurodegenerative diseases [22,23].

**Figure 3:**
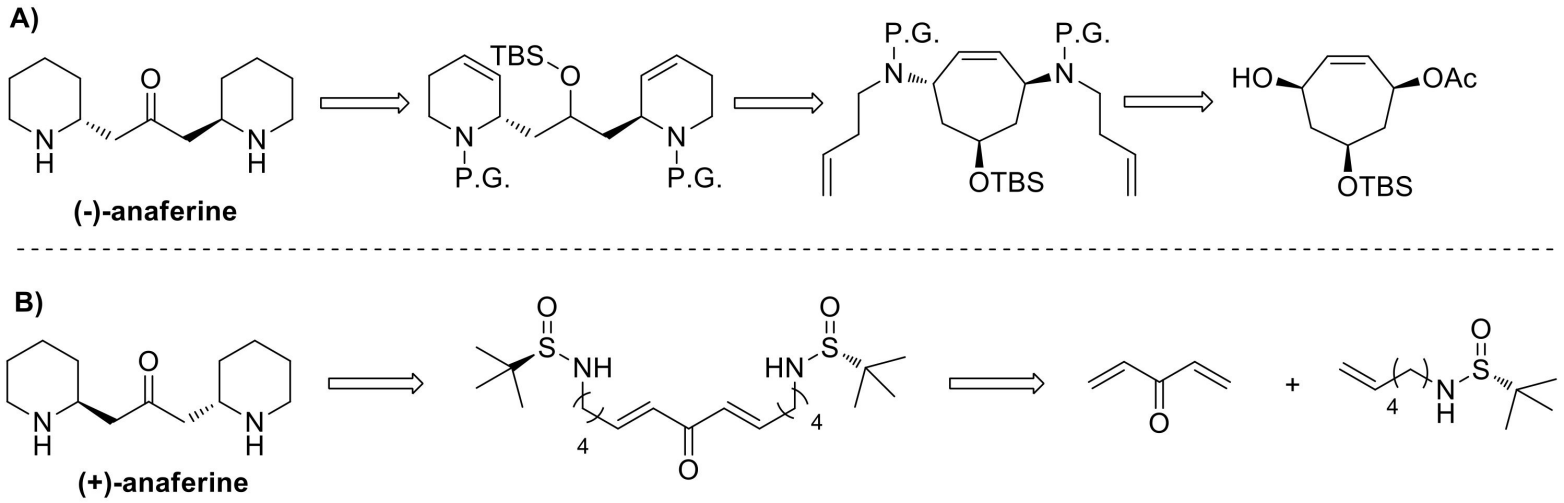
(**A**) Retrosynthetic pathway for the synthesis of (-)-anaferine, reported by Stapper and Blechert; (**B**) Retrosynthetic pathway for the synthesis of (+)-anaferine, reported by del Pozo and co-workers.

The last total synthesis of (–)-anaferine was accomplished in 2002 by Stapper and Blechert [24], while del Pozo et al. recently reported the preparation of the (+)-enantiomer [25] (Figure 3).

Stapper and Blechert’s strategy took advantage of an enantiopure cycloheptene precursor, accessed in five steps from the commercially available tropone. Then, after its conversion into the corresponding diamine, a tandem ring rearrangement metathesis, consisting in a ring-opening metathesis, followed by two ring-closing metatheses, afforded the fundamental carbon skeleton, that was transformed into the final product upon reduction of the double bonds, cleavage of the protecting groups and oxidation of the secondary alcohol to ketone (Figure 3A).

On the other hand, del Pozo’s approach involved the double use of *N*-sulfinyl amines, as both chiral auxiliaries and nucleophiles in an intramolecular aza-Michael reaction, as appreciable in Figure 3B. A bidirectional cross metathesis (CM), between the required sulfinyl amine and divinyl ketone gave an intermediate that underwent a double intramolecular aza-Michael reaction, forming the bis-piperidine core and generating at the same time the two stereocenters. Acidic cleavage of the sulfinyl groups led to (+)-anaferine synthesis completion.

## 2. Results and Discussion

**Scheme 1:**
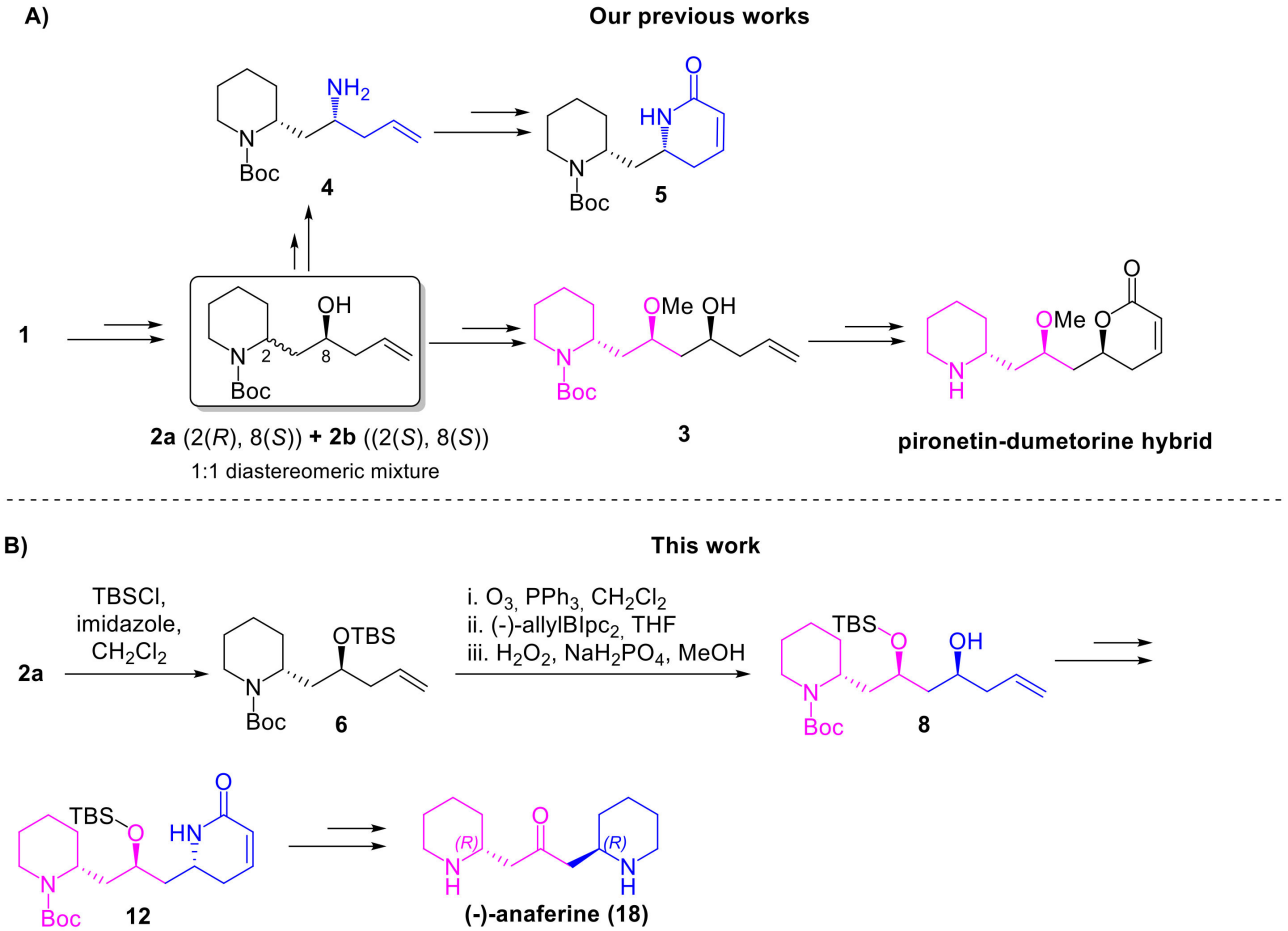
(**A**) Forward synthetic pathway for the generation of the key intermediates **3** and **5**, starting from 2-piperidine ethanol. (**B**) Planning of (-)-anaferine forward synthetic pathway.

The idea of enriching our DOS library with (–)-anaferine was suggested by compound **3** (Scheme 1A), previously obtained in our laboratory as an intermediate in the synthesis of the pironetin-dumetorine hybrids shown in Figure 2 [15]. Compound **3** is characterised by a piperidine core, deriving from the 2-piperidine ethanol, by a 2-methoxy propane bridge and by a homoallylic alcohol. The synthesis of **3**, starting from 2-piperidine ethanol is summarized in Scheme 1A, and involves as key step the conversion of **1** into the homoallylic alcohol **2a.** Notably, **2a** was recently exploited also for the synthesis of an α,β-unsaturated lactam (compound **5**), as appreciable again in Scheme 1A [18]. Therefore, comparing the two synthetic routes, it’s possible to deduce the genesis of our synthetic plan. In fact, we reasoned that an analogue (**8**) of compound **3**, bearing an O-silylether instead of the methoxy group, could have been easily obtained starting from **2a**, taking advantage of the same synthetic strategy. The homoallylic alcohol of **8** could be then converted into lactam **12**, exploiting the same approach employed for the synthesis of **5** from **2a**. The synthesis of (–)-anaferine could be completed reducing the *α,β*-unsaturated lactam of **12** to the corresponding piperidine, and oxidizing the secondary alcohol to ketone, as depicted in the forward synthetic Scheme 1B. Of course, the synthesis of (–)-anaferine requires a strict control over the stereochemistry, in order to access the correct *(R,R*)-enantiomer. To this extent, two asymmetric Brown allylations were considered.

The proposed synthetic strategy started from the homoallylic alcohol **2a**, previously obtained in our research group from **1** in three steps, involving the protection of the piperidinic nitrogen, oxidation of the alcohol to aldehyde and its asymmetric allylation under Brown’s conditions, leading to two diastereomeric alcohols **2a** and **2b** [18] (Scheme 1A). The relative *anti*-configuration of **2a** was established by X-ray analysis, while the desired absolute (*R,S*)-configuration resulted from the use of the proper allylating agent enantiomer (in this case the (–)-B-allyl diisopinocampheylborane). In this way, **2a** was obtained with an *e.e.* = 84% (Supplementary Information, Figure S1) and was then protected at the hydroxyl group as *tert*-butylsilyl ether (**6**).

**Figure 4:**
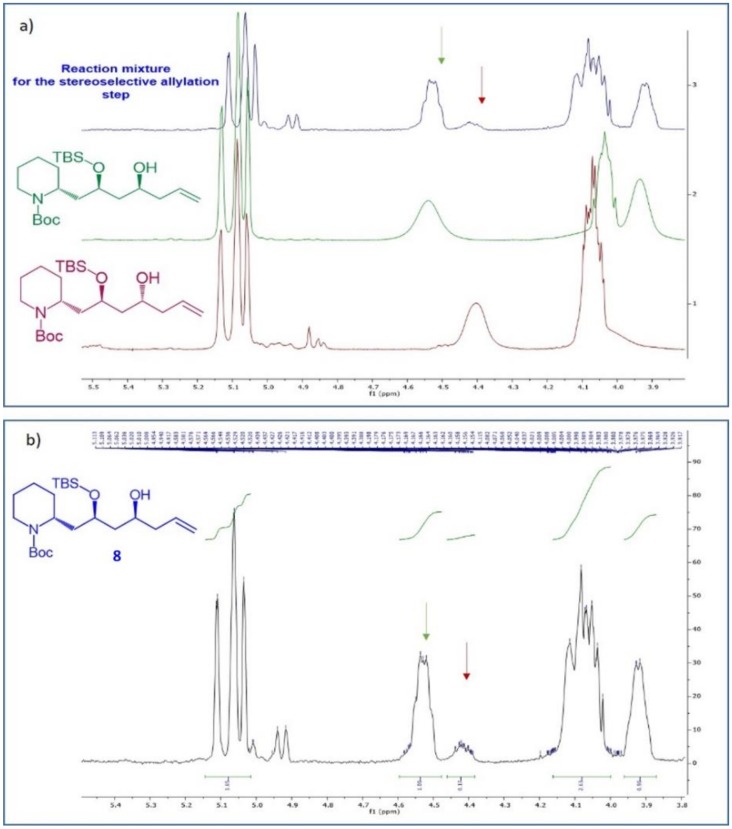
^1^H-NMR determination of the *d.r.* for the asymmetric allylation of aldehyde **7** (400 MHz, deuterated benzene). (**a**) ^1^H-NMR spectra of the reaction mixture for the asymmetric allylation (in blue) and of the two single diastereomers **8** and **8b** (green and red). In the first spectrum the presence of a major and a minor diastereomers is appreciable, as indicated by the two arrows. (**b**) elaboration of the blue spectrum of Figure 4a, showing the integrals exploited for the *d.r.* determination.

Oxidative cleavage of the double bond afforded aldehyde **7**, that was allylated again exploiting a Brown allylation. Also in this case, the stereocontrol was exerted mainly by the chirality of the Ipc (isopinocampheyl) substituents on the borane, rather than by the stereocenters already present in the substrate. The use of (–)-B-allyl diisopinocampheylborane, secured the formation of the desired stereoisomer of compound **8**, that was accessed with a *d.r.*: 85:15. The diastereomeric ratio was determined through ^1^H-NMR analysis. In fact, the two possible diastereomers **8** and **8b**, characterized by an opposite configuration at the C-10 stereocenter, were independently synthesised. Their ^1^H-NMR spectra in CDCl_3_ were almost completely superimposable, rendering impossible the determination of the diastereomeric composition of the reaction mixture obtained under asymmetric conditions. However, we realized that using deuterated benzene a satisfactory signals separation for the two single diastereomers was achieved (Figure 4a, green and red spectra). In this way, once identified the signals of each diastereomers, the *d.r.* of the asymmetric reaction (blue spectrum in Figure 4a) was calculated on the basis of signals integrals, as reported in Figure 4b.

With the correct stereoisomer of compound **8** in our hands, we proceeded with the following step, that was a Mitsunobu reaction in the presence of diphenylphosphorylazide, that converted the alcohol into the corresponding azide **9**, with inversion of configuration. In this way, the correct (*R*)-configuration of C-10 stereocenter was accessed. Azide reduction under mild Staudinger conditions afforded homoallylic amine **10**. Acylation with the commercially available acryloyl chloride, followed by a ring closing metathesis, led to the formation of the desired α,β-unsaturated lactam **12**. As reported in our previous studies, Umicore M73 SIMes catalyst proved to be the most efficient to favour the ring enclosure [18,26]. In this way, the fundamental (–)-anaferine carbon skeleton, characterised by two nitrogen-containing hexacycles, connected by the 2-hydroxypropane linker and maintaining the (*R,R*)-configuration of the two key stereocenters, was secured. The next goal was the reduction of the α,β-unsaturated lactam into the corresponding piperidine. First of all, the double bond was selectively reduced through catalytic hydrogenation in the presence of Pd/C. Then, the amidic nitrogen of **13** was protected as Boc [27] and the piperidone was reduced to piperidine **15,** using BH_3_·SMe_2_. This mild reducing agent was preferred to the most common LiAlH_4_, to avoid the conversion of the two Boc protecting groups into methyl amines [28]. The TBS protecting group was selectively cleaved using TBAF, affording the known intermediate **16**, reported in Blechert and Stapper’s synthesis of (–)-anaferine [24].

**Scheme 2:**
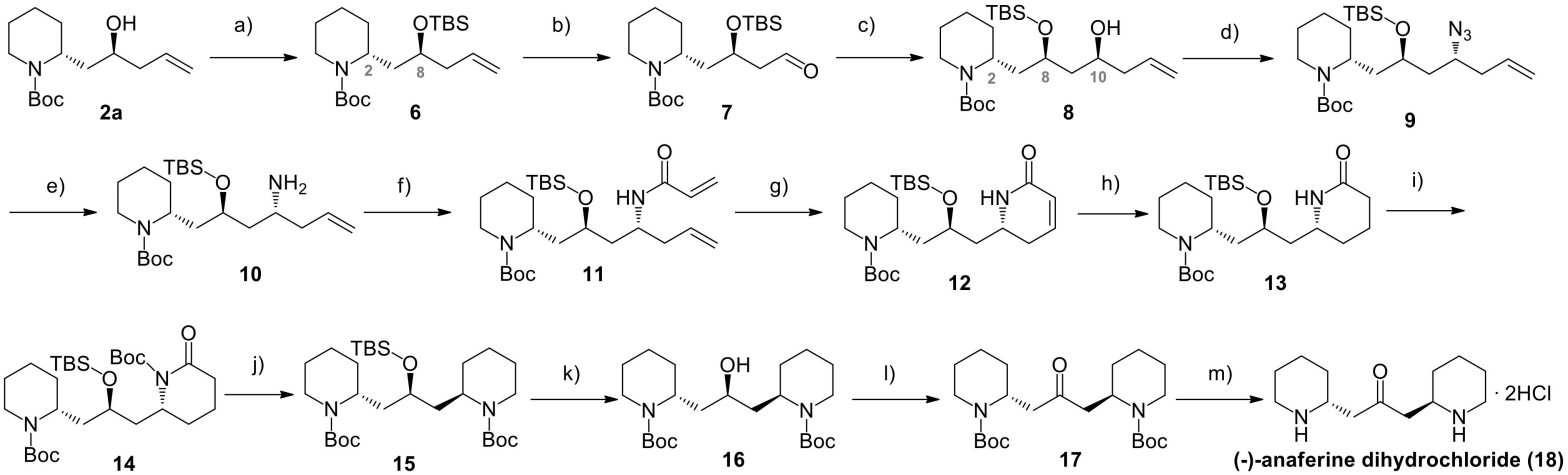
*Reagents and conditions*: (**a**) TBSCl, imidazole, CH_2_Cl_2_, 0 °C to r.t., 5 h, 90%; (**b**) O_3_, PPh_3_, CH_2_Cl_2_, −78 °C, to r.t., 3 h, 60%; (**c**) (–)-allyl-BIpc_2_, THF, −78 °C to r.t., 16 h, then H_2_O_2_, NaH_2_PO_4_, MeOH, 0.5 h, 92%, (**d**) PPh_3_, DIAD, DPPA, THF, r.t., 16 h, 85%; (**e**) PPh_3_, THF/H_2_O, 40 °C, 8 h, 80%; (**f**) acryloyl chloride, TEA, CH_2_Cl_2_, 0 °C to r.t., 5 h, 88%; (**g**) M73 SIMes cat. (1 mol %) CH_2_Cl_2_, 50 °C, 5 h, 71%; (**h**) H_2_, Pd/C (10% wt), MeOH, r.t., 16h, quant.; (**i**) Boc_2_O, TEA, DMAP, CH_2_Cl_2_, r.t., 48 h, 82%; (**j**) BH_3_ SMe_2_, THF, 0 °C, 5 h, 76%; (**k**) TBAF, THF, −15 °C to 0 °C, 16 h, 71%; (**l**) DMP, CH_2_Cl_2_, r.t., 16 h, quant.; (**m**) HCl 4M in dioxane, CH_2_Cl_2_, 0 °C to r.t., 16 h, quant.

The use of TBAF instead of acid conditions was fundamental to preserve the two Boc protecting groups: this was a mandatory requirement, because two basic nitrogen atoms would have interfered with the following oxidation of the hydroxyl group to ketone. Dess-Martin periodinane (DMP) was employed instead of pyridinium chlorochromate, that was the oxidizing agent of choice in the previously reported synthesis. DMP is a less toxic reagent, easier to handle and that gave, after a basic work-up, the desired product **17** in quantitative yield and without need of further purification. Finally, the cleavage of the two Boc protecting groups in acid environment, led to the desired (-)-anaferine dihydrochloride (**18**), whose identity was confirmed comparing analytical and spectroscopic properties with data reported in literature. The whole synthetic route is depicted in Scheme 2.

In conclusion, 2-piperidine ethanol proved to be once again a flexible synthon for the obtainment of diversified piperidine-containing compounds. In this work, the collection of derivatives previously synthesised in our research group was enriched with (–)-anaferine, that was accessed as a new branching of our diversity-oriented approach, with an overall 9% yield over 13 steps. Notably, this strategy could open the possibility of affording interesting analogues of piperidine-based marine and plant-deriving natural products, characterised by a polyketide side chain in C-2 position.

## 3. Experimental Section

### 3.1. General Remarks

Unless otherwise stated, reagents and solvents were purchased from Sigma Aldrich (St. Louis, MO, USA), Fluorochem (Zentek, Milano, Italy) or TCI (Zentek, Milano, Italy) and used without further purification. Unless otherwise stated, all reactions were carried out in oven-dried glassware and dry solvents, under nitrogen atmosphere and were monitored by thin layer chromatography (TLC) on silica gel (precoated 60F254 plates, Merck, (Darmstadt, Germany, with detection by UV light (254 nm) or by solutions of potassium permanganate stain, or ninhydrin, or *p*-anisaldehyde stain. Flash chromatography was performed using silica gel (240−400 mesh, Merck) as stationary phase.

^1^H-NMR spectra were recorded on an Avance (400 MHz) spectrometer (Bruker, Billerica, MA, USA) and are reported relative to residual CDCl_3_ or CD_3_OD. ^13^C-NMR spectra were recorded on the same instruments (100 MHz) and are reported relative to residual CDCl_3_ or CD_3_OD. All 1D and 2D NMR spectra (Bruker, Billerica, MA, USA) were collected using the standard pulse sequences available with Bruker Topspin 1.3. Chemical shifts (δ) for proton and carbon resonances are quoted in parts per million (ppm) relative to tetramethylsilane (TMS), used as an internal standard. Data for ^1^H-NMR are reported as follows: chemical shift (δ/ppm) (multiplicity, coupling constant (Hz), integration). Multiplicities are reported as follows: s = singlet, d = doublet, t = triplet, m = multiplet, bs = broad singlet. Data for ^13^C-NMR are reported in terms of chemical shift (δ/ppm).

Mass spectra were registered exploiting the electrospray ionisation (ESI) technique on a Q-Tof micro mass spectrometer (Waters, Milford, MA, USA).

Specific rotation values were measured on a P-1030 polarimeter (Jasco, Europe, Cremella, LC, Italy), using 1 mL cells, with path length of 10 cm. Measures were collected at 20–25 °C, using sodium D line wavelength λ = 589 nm.

### 3.2. Synthesis of tert-butyl (2R)-2-[(2S)-2-[(tert-butyldimethylsilyl)oxy]pent-4-en-1-yl]piperidine- 1-carboxylate (***6***)

TBSCl (0.574 g, 3.81 mmol) was added to a solution of **2a** (0.790 g, 2.93 mmol) and imidazole (0.400 g, 5.86 mmol) in anhydrous CH_2_Cl_2_ (15 mL), previously cooled at 0 °C. The reaction mixture was then diluted with CH_2_Cl_2_ and washed with brine. The aqueous layer was extracted with CH_2_Cl_2_ and the collected organic phases were dried over Na_2_SO_4_, filtered and concentrated under vacuum. The crude product was purified by column chromatography on silica gel (hexane: EtOAc = 9:1), to give **6** (1.010 g, 90%) as a yellow oil. [α]_D_^20^: +16.9 (*c* = 0.62, CHCl_3_). ^1^H-NMR (CDCl_3_) δ 5.95−5.65 (m, 1H), 5.14–4.94 (m, 2H), 4.36–4.16 (m, 1H), 4.07−3.87 (m, 1H), 3.67 (dq, *J* = 7.6, 5.2 Hz, 1H), 2.86–2.63 (m, 1H), 2.50–2.31 (m, 1H), 2.31–2.16 (m, 1H), 1.83 (m, 1H), 1.75–1.47 (m, 7H), 1.46 (s, 9H), 0.98−0.79 (m, 9H), 0.07 (s, 3H), 0.06 (s, 3H). ^13^C-NMR (CDCl_3_) δ 155.0, 135.3, 117.1, 79.4, 69.5, 47.5, 41.2, 39.0, 36.1, 36.8, 29.1, 28.7 (3 CH_3_), 26.0, 25.8 (3 CH_3_), 19.3, −4.3 (2 CH_3_). MS (ESI) *m/z* 406.31 [M + Na]^+^ (calcd. for C_21_H_41_NO_3_SiNa, 406.27).

### 3.3. Synthesis of tert-butyl (2R)-2-[(2R)-2-[(tert-butyldimethylsilyl)oxy]-4-oxobutyl]piperidine- 1-carboxylate (***7***)

Ozone was fluxed through a solution of **6** (1.000 g, 2.61 mmol) in CH_2_Cl_2_ (30 mL), cooled at −78 °C. After 1 h, the residual ozone was removed from the solution fluxing nitrogen for 10 min. PPh_3_ (1.026 g, 3.91 mmol) was added at −78 °C, then the reaction mixture was stirred for 2 h at r.t.. The solvent was removed under vacuum and the residue was purified by column chromatography on silica gel (hexane: EtOAc = 9:1), affording **7** (0.603 g, 60%) as a yellow oil. [α]_D_^20^: +22.2 (*c* = 0.52, CHCl_3_). ^1^H-NMR (CDCl_3_) δ 9.77 (s, 1H), 4.36−4.23 (m, 1H), 4.13 (d. quint., *J* = 11.5, 4.2 Hz, 1H), 4.07−3.95 (m, 1H), 2.76 (t, *J* = 12.2 Hz, 2H), 2.53 (ddd, *J* = 15.6, 7.0, 3.2 Hz, 1H), 2.14−1.97 (m, 1H), 1.56 (dd, *J* = 29.8, 11.2 Hz, 6H), 1.44 (s, 9H), 1.41−1.32 (m, 1H), 0.86 (s, 9H), 0.07 (s, 3H), 0.04 (s, 3H). ^13^C- NMR (CDCl_3_) δ 202.3, 155.0, 79.7, 66.2, 50.4, 47.4, 41.5, 38.2, 29.5, 28.6 (3 CH_3_), 25.9 (3 CH_3_), 25.8, 19.4, 18.1, −4.19, −4.60. MS (ESI) *m/z* 408.38 [M + Na]^+^ (calcd. for C_20_H_39_NO_4_SiNa, 408.25).

### 3.4. Synthesis of (R)-tert-butyl 2-((2R,4S)-2-((tert-butyldimethylsilyl)oxy)-4-hydroxyhept-6-en-1-yl) piperidine-1-carboxylate (***8***)

(-)-DIP-Cl (1.292 g, 4.03 mmol) was dissolved in anhydrous THF (16 mL) and cooled at −78 °C. Allylmagnesium bromide (1.0 M solution, 4.14 mL, 4.14 mmol) was added dropwise. The reaction mixture was stirred for 1 h at −5 °C and then the white solid was allowed to precipitate. **7** (0.914 g, 2.37 mmol) was dissolved in anhydrous THF (8 mL) and added to the supernatant solution of the previous mixture, cooled at −78 °C. After 1 h, the reaction was warmed to r.t. and stirred overnight. The mixture was concentrated and a saturated solution of NaH_2_PO_4_ (14mL) was added, followed by MeOH (14 mL) and H_2_O_2_ 35% (7mL). The mixture was stirred for 30 min; a saturated solution of NaHCO_3_ was added. The layers were separated and the aqueous one was extracted with Et_2_O. The combined organic layers were washed with brine, dried over Na_2_SO_4_, filtered and concentrated under vacuum. The crude product was purified by column chromatography on silica gel (hexane: EtOAc = 8:2, second column CH_2_Cl_2_, then CH_2_Cl_2_: MeOH = 95:5), to give **8** (0.932 g, 92%) as light yellow oil. [α]_D_^20^: +3.1 (*c* = 0.63, CHCl_3_). ^1^H-NMR (CDCl_3_) δ 5.86 (m, 1H), 5.17−5.02 (m, 2H), 4.36−4.20 (m, 1H), 4.15−3.91 (m, 1H), 3.91−3.76 (m, 2H), 2.78 (t, *J* = 12.7 Hz, 1H), 2.23 (m, 2H), 2.12−1.98 (m, 1H), 1.98−1.77 (m, 1H), 1.72−1.48 (m, 7H), 1.45 (s, 9H), 1.43−1.38 (m, 1H), 0.89 (s, 9H), 0.10 (s, 6H). ^13^C-NMR (CDCl_3_) δ 155.1, 135.2, 117.4, 79.6, 70.7, 70.1, 47.4, 43.7, 42.4, 39.2 (2 CH_2_), 30.0, 28.7 (3CH_3_), 26.0 (3CH_3_), 25.8, 19.4, 18.4, −3.9, −4.5. MS (ESI) *m/z* 450.79 [M + Na]^+^ (calcd. for C_23_H_45_NO_4_SiNa, 450.30).

### 3.5. Synthesis of tert-butyl(R)-2-((2R,4R)-4-azido-2-((tert-butyldimethylsilyl)oxy)hept-6-en-1-yl) piperidine-1-carboxylate (***9***)

**8** (0.932 g, 2.18 mmol) and PPh_3_ (0.687 g, 2.62 mmol) were dissolved in anhydrous THF (18 mL) and cooled at 0 °C. DIAD (0.52 mL, 2.62 mmol) was added dropwise. The reaction mixture was stirred for 10′ at 0 °C and then DPPA (0.57 mL, 2.62 mmol) was added dropwise. The reaction was warmed to r.t. and stirred overnight. The mixture was concentrated, and the crude product was purified by column chromatography on silica gel (hexane: EtOAc = 97:3), to give **9** (0.839 g, 85%) as light yellow oil. [α]_D_^20^: −29.7 (*c* = 0.54, CHCl_3_). ^1^H-NMR (CDCl_3_) δ 5.82 (ddt, *J* = 17.1, 10.1, 7.0 Hz, 1H), 5.21−5.08 (m, 2H), 4.36−4.22 (m, 1H), 4.09−3.94 (m, 1H), 3.80 (m, 1H), 3.66−3.54 (m, 1H), 2.77 (t, *J* = 13.1 Hz, 1H), 2.35 (m, 2H), 2.10−1.98 (m, 1H), 1.80−1.66 (m, 1H), 1.64−1.53 (m, 5H), 1.51 (m, 1H), 1.47 (s, 9H), 1.44−1.36 (m, 2H), 0.89 (s, 9H), 0.09 (s, 3H), 0.08 (s, 3H). ^13^C-NMR (CDCl_3_) 154.1, 134.1, 118.4, 79.4, 67.2, 58.6, 47.3, 41.1, 39.6, 38.6 (2 CH_2_), 29.8, 28.6 (3 CH_3_), 26.1 (3 CH_3_), 25.8, 19.4, 18.2, −3.82, −4.69. MS (ESI) *m/z* 475.31 [M + Na]^+^ (calcd. for C_23_H_44_N_4_O_3_SiNa, 475.31).

### 3.6. Synthesis of (R)-tert-butyl 2-((2S,4R)-4-amino-2-((tert-butyldimethylsilyl)oxy)hept-6-en-1-yl) piperidine-1-carboxylate (***10***)

Compound **9** (0.821 g, 1.81 mmol) and PPh_3_ (0.951 g, 3.63 mmol) were dissolved in a mixture of THF and H_2_O (10:1, 25 mL) and heated at 40 °C for 8 h. At that point, water was added (10.4 mL). The layers were separated and the aqueous one was extracted with Et_2_O. The combined organic layers were washed with brine, dried over Na_2_SO_4_, filtered and concentrated under vacuum. The crude product was purified by column chromatography on silica gel (CH_2_Cl_2_: MeOH = 95:5), to give **10** (0.601 g, 78%) as light yellow oil. [α]_D_^20^: +6.8 (*c* = 0.46, CHCl_3_). ^1^H-NMR (CDCl_3_) δ 5.79 (ddt, *J* = 17.2, 10.2, 7.1 Hz, 1H), 5.31−4.94 (m, 2H), 4.28–4.12 (m, 1H), 4.08–3.90 (m, 1H), 3.87–3.80 (m, 1H), 3.10–3.05 (m, 1H), 2.90−2.59 (m, 1H), 2.40–2.20 (m, 2H), 2.09−2.01 (m, 1H), 1.76−1.48 (m, 7H), 1.45 (s, 9H), 1.40−1.30 (m, 2H), 0.96−0.75 (m, 9H), 0.10–−0.15 (m, 6H). ^13^C-NMR (CDCl_3_) δ 155.3, 135.3, 117.9, 79.6, 68.4 (2 CH), 47.8, 42.5 (2 CH_2_), 39.00, 37.8, 29.8, 28.7 (3 CH_3_), 26.0 (3 CH_3_), 25.8, 19.4, 18.1, −4.17, −4.47. MS (ESI) *m/z* 427.63 [M + H]^+^ (calcd. for C_23_H_47_N_2_O_3_Si, 427.33).

### 3.7. Synthesis of (R)-tert-butyl 2-((2S,4R)-4-acrylamido-2-((tert-butyldimethylsilyl)oxy) hept-6-en- 1-yl) piperidine-1-carboxylate (***11***)

Compound **10** (0.558 g, 1.31 mmol) was dissolved in anhydrous CH_2_Cl_2_ (5.5 mL) and cooled at 0 °C. Anhydrous TEA (547 μL, 3.92 mmol) was added and the reaction was stirred for 10 min. Acryloyl chloride (160 μL, 1.96 mmol) was added dropwise; the mixture was warmed to r.t. and stirred for 5 h. At that point, cold water (0 °C) was added. The layers were separated and the aqueous one was extracted with CH_2_Cl_2_. The combined organic layers were washed twice with a saturated solution of NH_4_Cl, dried over Na_2_SO_4_, filtered and concentrated under vacuum. The crude product was purified by column chromatography on silica gel (CH_2_Cl_2_: MeOH = 95:5), to give **11** (0.548 g, 88%) as a brown oil. [α]_D_^20^: −8.1 (*c* = 0.42, CHCl_3_). ^1^H-NMR (CDCl_3_) δ 6.42 (bs, 1H), 6.29−6.03 (m, 2H), 5.81 (ddt, J = 19.2, 9.6, 7.2 Hz, 1H), 5.65−5.51 (m, 1H), 5.16−4.94 (m, 2H), 4.22−4.09 (m, 1H), 4.05−3.91 (m, 2H), 3.77−3.60 (m, 1H), 2.86 (m, 1H), 2.53−2.30 (m, 1H), 2.14−2.02 (m, 1H), 1.80 (t, J = 12.8 Hz, 1H), 1.66−1.53 (m, 6H), 1.45 (m, 11H), 1.31 (m, 1H), 0.91 (s, 9H), 0.02 (s, 6H). ^13^C-NMR (CDCl_3_) δ 165.3, 134.6, 131.7, 125.4, 117.4, 79.6, 66.8, 46.6, 39.3 (3CH_2_), 31.4, 30.5 (2CH_2_), 28.6 (3CH_3_), 25.9 (3CH_3_), 25.6, 19.3 (2 CH_2_), 17.9, −4.5 (2CH_3_). MS (ESI) *m/z* 503.71 [M + Na]^+^ (calcd. for C_26_H_48_N_2_O_4_SiNa, 503.33).

### 3.8. Synthesis of (R)-tert-butyl 2-((S)-2-((tert-butyldimethylsilyl)oxy)-3-((R)-6-oxo- 1,2,3,6- tetrahydro-pyridin -2-yl)propyl)piperidine-1-carboxylate (***12***)

Compound **11** (0.540 g, 1.12 mmol) was dissolved in anhydrous degassed CH_2_Cl_2_ (47 mL). A solution of Umicore catalyst 14% wt (59.0 mg, 0.01 mmol) in degassed CH_2_Cl_2_ (16.2 mL) was added dropwise and the reaction was stirred for 5 h at 50 °C. The reaction mixture was concentrated under vacuum. The crude product was purified by column chromatography on silica gel (CH_2_Cl_2_: MeOH = 98:2), to give **12** (0.364 g, 71%) as a light yellow oil. [α]_D_^20^: +46.2 (*c* = 0.66, CHCl_3_). ^1^H-NMR (CDCl_3_) δ6.67 (bs, 1H), 6.60−6.51 (m, 1H), 5.91−5.83 (m, 1H), 4.19 (m, 1H), 3.99−3.94 (m, 1H), 3.80−3.76 (m, 2H), 2.81−2.73 (m, 1H), 2.17−2.16 (m, 2H), 1.84-1.75 (m, 2H), 1.54−1.46 (m, 6H), 1.45 (s, 9H), 1.36−1.28 (m, 2H), 0.89 (s, 9H), 0.06 (s, 6H). ^13^C-NMR (CDCl_3_) δ 166.0, 155.5, 140.4, 125.2, 79.9, 68.2, 48.0 (2 CH), 31.5 (2 CH_2_), 30.0, 29.8, 28.6 (3 CH_3_), 26.0 (3 CH_3_), 25.9, 25.7, 19.3, 18.1, −4.3, −4.8. MS (ESI) *m/z* 475.52 [M + Na]^+^ (calcd. for C_24_H_44_N_2_O_4_SiNa, 475.30).

### 3.9. Synthesis of (R)-tert-butyl 2-((S)-2-((tert-butyldimethylsilyl)oxy)-3-((R)-6- oxopiperidin -2-yl)propyl) piperidine-1-carboxylate (***13***)

Compound **12** (0.205 g, 0.45 mmol) was dissolved in MeOH (10 mL); Pd/C 10% wt (0.205 g) was added, and the reaction mixture was put under H_2_ atmosphere and stirred overnight. The mixture was filtered over Celite, washed with MeOH and CH_2_Cl_2_ and concentrated to give **13** (0.206 g, quant.) as a dark yellow oil. [α]_D_^20^: +1.4 (*c* = 0.70, CHCl_3_). ^1^H-NMR (CDCl_3_) δ 6.73 (s, 1H), 4.23−4.19 (m, 1H), 4.00−3.96 (m, 1H), 3.82−3.77 (m, 1H), 3.59−3.54 (m, 1H), 2.78 (t, *J* = 13.2 Hz, 1H), 2.44−2.32 (m, 1H), 2.25 (ddd, *J* = 17.7, 11.1, 6.5 Hz, 1H), 2.07 (ddd, J = 14.6, 11.4, 3.7 Hz, 1H), 1.88 (m, 1H), 1.84−1.66 (m, 4H), 1.66−1.48 (m, 6H), 1.45 (s, 9H), 1.37 (m, 2H), 0.91 (s, 9H), 0.08 (s, 6H). ^13^C-NMR (CDCl_3_) δ 171.6, 155.2, 79.8, 68.6, 49.9, 47.7, 41.5, 39.2, 31.3, 30.3, 30.0, 29.9, 28.7 (3 CH_3_), 26.1 (3 CH_3_), 25.8, 20.6, 19.4, 18.2, −4.59, −4.91. MS (ESI) *m/z* 477.91 [M + Na]^+^ (calcd. for C_24_H_46_N_2_O_4_SiNa, 477.31).

### 3.10. Synthesis of (R)-tert-butyl 2-((R)-3-((R)-1-(tert-butoxy carbonyl)piperidin-2-yl)-2- ((tert-butyl-dimethylsilyl)oxy)propyl)-6-oxopiperidine-1-carboxylate (***14***)

Compound **13** (0.206 g, 0.45 mmol) was dissolved in anhydrous CH_2_Cl_2_ (1.5 mL) and cooled to 0 °C. TEA (190 μL, 1.36 mmol), DMAP (0.012 g, 0.09 mmol) and Boc_2_O (0.298 g, 1.36 mmol) were added. The mixture was stirred at r.t. for 48 h and concentrated under vacuum. The crude product was purified by column chromatography on silica gel (hexane: EtOAc = 7:3), to give **14** (0.204 g, 82%) as a dark yellow oil. [α]_D_^20^: +23.2 (*c* = 0.82, CHCl_3_). ^1^H-NMR (CDCl_3_) δ 4.48−4.33 (m, 1H), 4.23 (m, 1H), 4.08−3.92 (m, 1H), 3.78 (quint., *J* = 6.2 Hz, 1H), 2.77 (t, *J* = 12.7 Hz, 1H), 2.55−2.38 (m, 2H), 1.96−1.70 (m, 7H), 1.88- 1.56 (m, 5H), 1.52 (s, 9H), 1.44 (s, 9H), 1.31−1.19 (m, 2H), 0.88 (s, 9H), 0.07 (s, 6H). ^13^C-NMR (CDCl_3_) δ 171.3, 154.8, 152.8, 82.9, 79.2, 68.5, 53.7, 47.3, 41.6, 39.2, 36.7, 34.1, 29.7, 28.6 (3 CH_3_), 28.0 (3 CH_3_), 26.7, 25.9 (3 CH_3_), 25.6, 19.2, 17.9, 17.1, −4.3 (2 CH_3_). MS (ESI) *m/z* 577.48 [M + Na]^+^ (calcd. for C_29_H_54_N_2_O_6_SiNa: 577.36).

### 3.11. Synthesis of (2R,2′R)-di-tert-butyl 2,2′-(2-((tert-butyldimethylsilyl)oxy)propane- 1,3-diyl) bis (piperidine-1-carboxylate) (***15***)

Compound **14** (0.170 g, 0.31 mmol) was dissolved in anhydrous THF (7.5 mL) and cooled to 0 °C. BH_3_∙SMe_2_ (68 μL, 0.67 mmol), was added dropwise. The mixture was stirred at r.t. for 5h and quenched with a saturated solution of NH_4_Cl. The layers were separated, and the organic one was extracted with Et_2_O. The combined organic layers were dried over Na_2_SO_4_, filtered and concentrated under vacuum. The crude product was purified by column chromatography on silica gel (hexane: EtOAc = 8:2), to give **15** (0.126 g, 76%) as a yellow oil. [α]_D_^20^: +28.0 (*c* = 0.48, CHCl_3_). ^1^H-NMR (CDCl_3_) δ 4.40–4.24 (m, 2H), 4.00–3.87 (m, 2H), 3.72–3.61 (m, 1H), 2.84–2.71 (m, 2H), 1.94–1.63 (m, 6H), 1.60–1.51 (m, 6H), 1.45 (s, 18H), 1.39–1.34 (m, 4H), 0.89 (s, 9H), 0.06 (s, 6H). ^13^C-NMR (CDCl_3_) δ 154.9 (2 Cq), 79.1 (2 Cq), 68.7, 47.4 (2 CH), 39.5 (2 CH_2_), 37.6 (2 CH_2_), 29.68 (2 CH_2_), 28.5 (6 CH_3_), 25.7 (3 CH_3_), 25.6 (2 CH_2_), 19.1 (2 CH_2_), 18.01, −4.2, −4.4. MS (ESI) *m/z* 563.99 [M + Na]^+^ (calcd. for C_29_H_56_N_2_O_5_SiNa, 563.38).

### 3.12. Synthesis of (2R,2′R)-di-tert-butyl 2,2′-(2-hydroxypropane-1,3- diyl)bis(piperidine-1-carboxylate) (***16***)

Compound **15** (0.112g, 0.21 mmol) was dissolved in anhydrous THF (1.5 mL) and cooled to −15 °C. TBAF 1.0 M in THF (620 μL, 0.62 mmol), was added dropwise. The mixture was stirred for 16h, slowly warming to 0 °C. The mixture was quenched with a saturated solution of NH_4_Cl. The layers were separated, and the organic one was extracted with Et_2_O. The combined organic layers were dried over Na_2_SO_4_, filtered and concentrated under vacuum. The crude product was purified by column chromatography on silica gel (hexane: EtOAc = 85:15), to give **16** (0.090 g, 71%) as a white solid. [α]_D_^20^: +43.3 (*c* = 0.53, CHCl_3_). Ref: [α]_D_^20^: +41.4 (*c* = 0.72, CHCl_3_). ^1^H-NMR (CDCl_3_) δ = 4.28−4.52 (m, 2 H), 3.70−3.98 (m, 2 H), 3.08−3.34 (m, 1 H), 2.60−2.92 (m, 2 H), 1.80−2.38 (m, 2 H), 1.00−1.74 (m, 14 H), 1.42 (s, 18 H). ^13^C-NMR (CDCl_3_) δ = 156.5, 155.0, 79.9, 79.0, 64.7, 47.4, 46.3, 39.3, 39.0, 37.0, 36.7, 29.7, 29.4, 28.5 (3 CH_3_), 28.3 (3 CH_3_), 25.6, 25.5, 19.1 (detected signals). MS (ESI) *m/z* 449.71 [M + Na]^+^ (calcd. for C_23_H_42_N_2_O_5_Na, 449.30).

### 3.13. Synthesis of (2R,2′R)-di-tert-butyl 2,2′-(2-oxopropane-1,3-diyl)bis(piperidine-1- carboxylate) (***17***)

Compound **16** (0.043 g, 0.10 mmol) was dissolved in anhydrous CH_2_Cl_2_ (1 mL) at 0 °C, and DMP (50.7 mg, 0.12 mmol) was added. The mixture was stirred for 16h at r.t. The mixture was concentrated; a 10% solution of NaOH was added and the aqueous phase was extracted with Et_2_O. The combined organic layers were washed with NaOH (10%) and H_2_O. The combined organic layers were dried over Na_2_SO_4_, filtered and concentrated under vacuum, to give **17** (0.042 g, quant.) as a colourless solid. [α]_D_^20^: +19.9 (*c* = 0.77, CHCl_3_). ^1^H-NMR (CDCl_3_) δ = 4.60−4.76 (m, 2 H), 3.82−4.02 (m, 2H), 2.50−2.86 (m, 6 H), 1.20−1.71 (m, 12 H), 1.41 (s, 18 H). ^13^C-NMR (CDCl_3_) δ 207.2, 154.8 (2 Cq), 79.6 (2 Cq), 47.3 (2 CH), 43.4 (2 CH_2_), 39.6 (2 CH_2_), 28.5 (6 CH_3_), 28.4 (2 CH_2_), 25.4 (2 CH_2_), 19.1 (2 CH_2_). MS (ESI) *m/z* 447.81 [M + Na]^+^ (calcd. for C_23_H_40_N_2_O_5_Na, 447.28).

### 3.14. Synthesis of 1,3-di((R)-piperidin-2-yl)propan-2-one (***18***)

Compound **17** (0.030 g, 0.07 mmol) was dissolved in CH_2_Cl_2_ (2 mL) and cooled to 0 °C. A 4M solution of HCl (75 μL, 0.21 mmol) in dioxane was added dropwise. The mixture was stirred for 16h at r.t. The mixture was concentrated to give (–)-anaferine dihydrochloride (**18**, 0.021 g, quant.) as a colourless solid, without need of further purification. [α]_D_^20^: −47.9 (*c* = 0.65, MeOH/H_2_O = 1:1). ^1^H NMR (CD_3_OD) δ = 3.50−3.61 (m, 2 H), 3.32−3.40 (m, 2 H), 2.88−3.05 (m, 6 H), 1.82−1.95 (m, 6 H), 1.00−1.74 (m, 6 H). ^13^C-NMR (CD_3_OD) δ = 205.0, 52.3 (2 CH), 44.8 (2CH_2_), 44.7 (2CH_2_), 28.3 (2CH_2_), 22.0 (2CH_2_), 21.6 (2CH_2_). MS (ESI) *m/z* 224.31 [C_13_H_24_N_2_O]^+^ (calcd. for C_13_H_24_N_2_O, 224.19).

## Supplementary Materials

Figure S1: Chiral HPLC analysis for the determination of the *e.e.* of compound **2a**.
