# Supplementary material for: Total Synthesis of (–)-Anaferine: A Further Ramification in a Diversity-Oriented Approach
Source: Molecules. 2020 Feb 27;25(5):1057. doi: 10.3390/molecules25051057 (PMC7179133; doi:10.3390/molecules25051057)
Supplement: Supplementary file 1 [file molecules-25-01057-s001.pdf]

Supplementary information

Total Synthesis of (-)-Anaferine: a Further Ramification in a Diversity-Oriented Approach

Elisa Bonandi [1,*], Giada Tedesco [1], Dario Perdicchia [1], and Daniele Passarella [1,*]

[1] Università degli Studi di Milano, Via Golgi 19, 20133, Milano (Italy); elisa.bonandi@unimi.it (E. B.), (G.T.), dario.perdicchia@unimi.it (D.P.)

* Correspondence: elisa.bonandi@unimi.it (E. B.); daniele.passarella@unimi.it; Tel.: +39 0250314081.

Figure S1. Chiral HPLC analysis for the determination of **2a** enantiomeric excess.

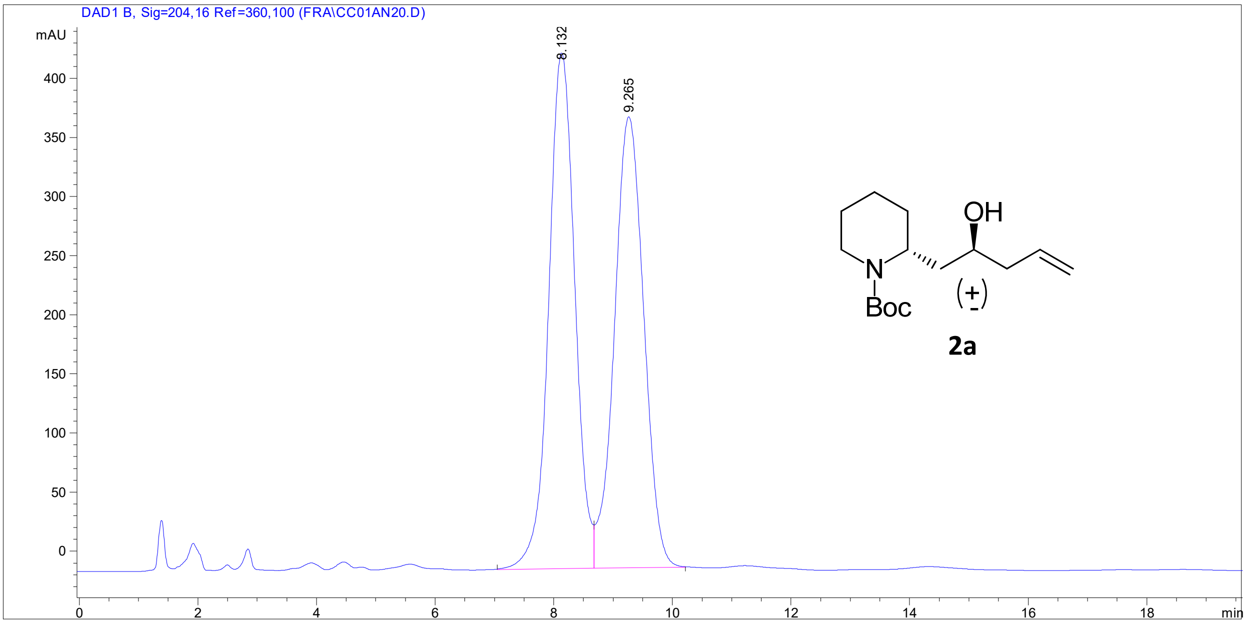

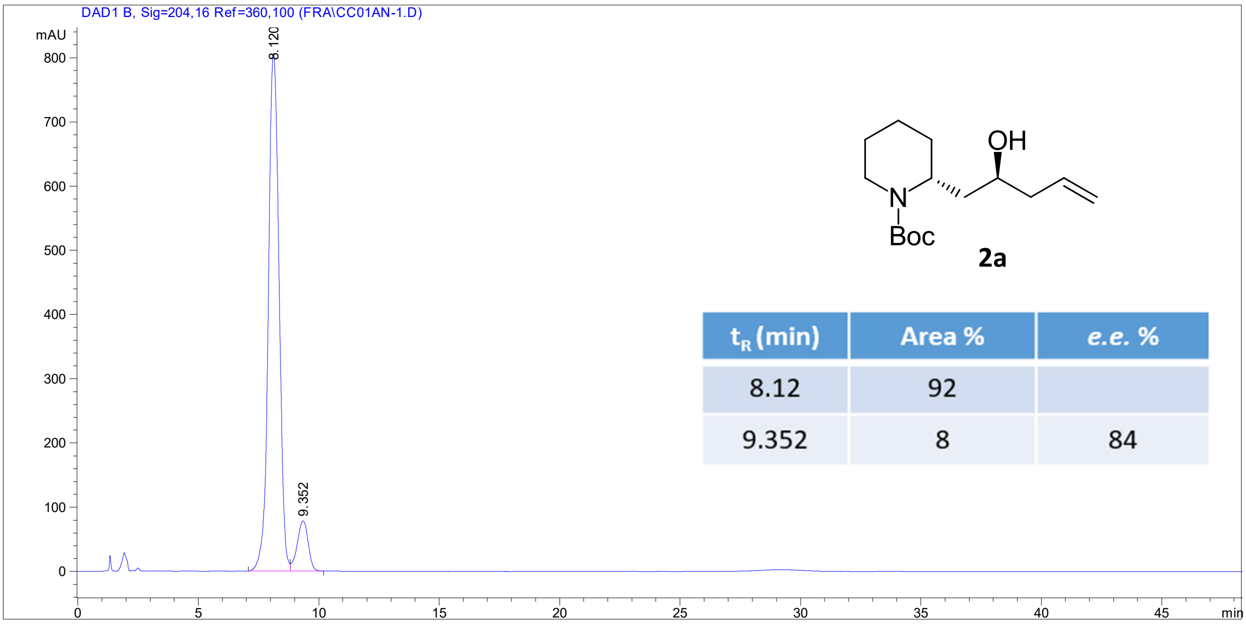

t_R (min)	Area %	*e.e.* %
8.12	92	
9.352	8	84

Figure S1. Chiral HPLC analysis for the determination of the *e.e.* of compound **2a**.
HPLC chromatogram of the racemic compound **2a** is reported on the top, while on the bottom the one of the enantiopure compound is appreciable. HPLC conditions: chiralcel AD-RH RP column, 1 mL/min, CH_3CN:H_2O = 35:65, 96 bar, λ: 204 nm.
